# Identification and validation of an anoikis-related lncRNA signature to predict prognosis and immune landscape in osteosarcoma

**DOI:** 10.3389/fonc.2023.1156663

**Published:** 2023-03-23

**Authors:** Jun-Song Zhang, Run-Sang Pan, Xiao-Bin Tian

**Affiliations:** ^1^ School of Clinical Medicine, Guizhou Medical University, Guiyang, China; ^2^ School of Basic Medicine, Guizhou Medical University, Guiyang, China; ^3^ Department of Orthopedics, The Affiliated Hospital of Guizhou Medical University, Guiyang, China

**Keywords:** osteosarcoma, anoikis, lncRNA, prognosis, immune

## Abstract

**Background:**

Anoikis is a specialized form of programmed apoptosis that occurs in two model epithelial cell lines and plays an important role in tumors. However, the prognostic value of anoikis-related lncRNA (ARLncs) in osteosarcoma (OS) has not been reported.

**Methods:**

Based on GTEx and TARGET RNA sequencing data, we carried out a thorough bioinformatics analysis. The 27 anoikis-related genes were obtained from the Gene Set Enrichment Analysis (GSEA). Univariate Cox regression and least absolute shrinkage and selection operator (LASSO) analysis were successively used to screen for prognostic-related ARLncs. To create the prognostic signature of ARLncs, we performed multivariate Cox regression analysis. We calculated the risk score based on the risk coefficient, dividing OS patients into high- and low-risk subgroups. Additionally, the relationship between the OS immune microenvironment and risk prognostic models was investigated using function enrichment, including Gene ontology (GO), Kyoto Encyclopedia of Genes and Genomes (KEGG), single-sample gene set enrichment analysis (ssGSEA), and GSEA analysis. Finally, the potential effective drugs in OS were found by immune checkpoint and drug sensitivity screening.

**Results:**

A prognostic signature consisting of four ARLncs (AC079612.1, MEF2C-AS1, SNHG6, and TBX2-AS1) was constructed. To assess the regulation patterns of anoikis-related lncRNA genes, we created a risk score model. According to a survival analysis, high-risk patients have a poor prognosis as they progress. By using immune functional analysis, the lower-risk group demonstrated the opposite effects compared with the higher-risk group. GO and KEGG analysis showed that the ARLncs pathways and immune-related pathways were enriched. Immune checkpoints and drug sensitivity analysis might be used to determine the better effects of the higher group.

**Conclusion:**

We identified a novel prognostic model based on a four-ARLncs signature that might serve as potential prognostic indicators that can be used to predict the prognosis of OS patients, and immunotherapy and drugs that may contribute to improving the overall survival of OS patients and advance our understanding of OS.

## Introduction

Osteosarcoma (OS) is the most common primary malignant bone tumor and is common in children and adolescents (1). It has an annual prevalence of three to four cases per million people, but it is an uncommon condition (2). Neoadjuvant chemotherapy is linked to a better 5-year overall survival in patients with limb osteosarcoma (3). Osteosarcoma generally develops in weight-bearing long bones, with the distal femur and proximal tibia being the most frequent sites (4). Compared with surgery and chemotherapy, a standard therapy for osteosarcoma has been developed; however, therapeutic advancement is still limited (5, 6). Osteosarcoma’s poor prognosis was strongly correlated with both its high recurrence rate and distant lung metastasis (7). Numerous studies built on clinical knowledge are looking into osteosarcoma therapeutic approaches. However, finding precise and efficient therapy targets for OS prognosis is challenging (8). Therefore, in order to develop new therapeutic strategies, it is crucial to investigate the regulatory mechanisms of genes related to osteosarcoma. lncRNAs, also known as transcripts, are ncRNAs that are longer than 200 nucleotides and do not code for proteins (9–11). Long non-coding RNAs (lncRNAs) participate in multiple biological processes and regulate physiological functions (12). Anoikis is a specialized form of programmed apoptosis that occurs in two model epithelial cell lines when cell adherence to the ECM is lost or cell–matrix interactions are disrupted (13, 14). Anoikis resistance is a property that cancer cells can acquire, allowing them to grow and spread from their primary sites to distant organs (15). By preventing detached cells from adhering to other substrates for abnormal proliferation, apoptosis plays a crucial role in protecting the organism (16). Numerous studies have demonstrated a connection between anoikis-related genes (ARGs) and the tumor metastasis cascade and cancer development (17)—for instance, human osteosarcoma cells become anoikis resistant by upregulating VEGF-A expression, which may promote angiogenesis and lung metastasis in OS (18). Numerous studies have revealed that lncRNA dysregulation is a significant factor in the modulation of anoikis resistance in a variety of cancers (19). lncRNAs have the ability to positively or negatively influence anoikis resistance (20). There are not many papers that analyze prognostic markers based on ARLncs in OS, even though many cancers have been shown to be related to anoikis (21–23). Thus, it is significant to focus on the relationship between integrated ARGs and OS clinical outcomes to investigate the therapeutics of OS. Using prognostic models, genes associated with necroptosis and apoptosis have been found to predict OS prognosis (24, 25). In this study, we developed a novel OS prognostic model to investigate anoikis-related lncRNA (ARLncs), which can be used to develop risk models and immunological features. We identified a robust ARLncs-based signature and exploited its clinical implications in OS patients. It is helpful for us to explore the prediction of how the tumor immunity microenvironment is regulated by the ARLncs signature, which has promising application possibilities.

## Methods

### Data acquisition

The gene expression and clinical information of 88 osteosarcoma patients were taken from the TARGET database (https://portal.gdc.cancer.gov/). From the GTEx database (https://gtexportal.org/), the gene expression information for 803 samples of normal muscular tissues was gathered. We excluded the samples that lacked sufficient information and divided the remaining samples into training and test groups of 42 each using a 1/1 ratio analysis. The expression data of gene for each sample were transformed into log2 (*x* + 1) to remove data differences caused by different platforms between the TCGA and GTEx databases. Batch-to-batch variation was then eliminated by combining two datasets into a single dataset using the combat function from the “sva” R package.

### Differentially expressed anoikis-related lncRNAs

With |log2FC| >1 and false discovery rate (FDR) <0.05, we use the R package “limma” to identify the lncRNA genes that are differentially expressed between 88 OS tissues and 803 normal tissues. The 27 anokis-related genes were collected from the gene set enrichment analysis (GSEA). According to Pearson’s correlation, the anoikis-related lncRNAs were found with Cor >0.4 and *P <*0.001 (26).

### Construction of anoikis-related lncRNA prognostic signatures

The “survival” package in R was used to identify lncRNAs associated with patient prognosis among the anoikis-related DE-lncRNAs by using uni-Cox regression with a *P*-value <0.05. To identify anoikis-related lncRNAs, we utilized 10-fold cross-validation and LASSO regression. Additionally, the anoikis-related lncRNA signature was chosen as the modeling gene after performing a multi-Cox regression analysis using the “glmnet” package to identify the optimum penalty value. The following formula calculates the risk score of OS patients:


$$\text{Risk score }=\;\sum_{\text{i}=1}^{\text{n}}\left({\text{lncRNAexp}}_{\text{i}}\;\times \;{\text{coef}}_{\text{i}}\right)$$

By computing the median risk score according to the algorithm, the OS patients were divided into low- and high-risk groups. The KM survival analysis of anoikis-related lncRNA signatures was built in the whole and in subgroups by the “survival” package in R software. Based on the timeROC R packages, the time-related and clinical-related ROC curves were built to investigate the predictive capacity of this gene signature. To determine if risk score can be seen as a factor influencing the prognosis of OS patients, the survival package in R was employed (27).

### Construction of nomogram, calibration, clinical forest, C-index, PCA, and t-SNE

Combined with risk scores, the nomogram was set up with the rms R package based on gender, age, tumor site, and metastasis. The C-index was established to evaluate the prediction bias of the nomogram. According to related R packages, we combined clinical data and risk scores to build a forest tree by uni- and multi-Cox regression analysis. To assess the efficacy of the risk model in OS patients in the low- and high-risk groups, principal component analysis (PCA) and t-distributed stochastic neighbor embedding (t-SNE) were constructed, respectively, on the risk prognosis in the entire, training, and test samples.

### Functional analysis, GSEA analysis, and immunological microenvironment analysis

In order to examine the underlying biological process, we determined the differential genes between the low-risk group and the high-risk group for volcano plot, heat map, and later Gene Ontology (GO) analysis. Additionally, GSEA was used to determine the KEGG pathways that were differentially expressed in the two groups. The KEGG gene set (c2.cp.kegg.v2022.1.Hs.symbols.gmt) can be obtained from the website (https://www.gsea‐msigdb.org/). The website served as the source for the KEGG gene set. *P*-value <0.05 was chosen as the cutoff for biological processes and pathways that were significantly enriched. Using R software’s limma, reshape2, and ggpubr packages, we examined the expression differences of immune cells and immune function between the low- and high-risk groups of the entire sample (28).

### Analysis of immune checkpoints and drug sensitivity

The “pRRophetic” package of R is commonly used to perform chemotherapeutic prediction (29, 30). The R package “pRRophetic” is utilized to investigate the difference in drug sensitivity among OS patients. Drug sensitivity analysis, using the standard of *P <*0.001, was used to identify whether the sensitivity of drugs was different in the risk prognosis model including all samples. We evaluate the expression of immune checkpoint-related genes in the low- and high-risk groups of OS patients to examine the impact of immunotherapy with the help of “reshap2”, “limma”, “ggplot2”, and “ggpubr” packages.

## Results

### Identification of a risk prognostic model 8in osteosarcoma

**Figure 1:**
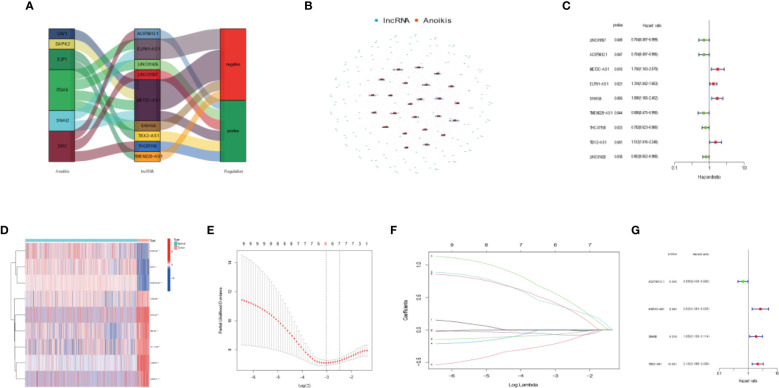
Developing a four anoikis-related long noncoding RNA (lncRNA) signature for osteosarcoma patients. **(A)** Sankey diagram highlighting the relationship between the anoikis genes and the anoikis-related lncRNAs. **(B)** Co-expression network showing the relationship between the expression of lncRNAs and that of Anoikis-associated genes. **(C)** Forest plot highlighting the univariate Cox regression analysis results. **(D)** Heatmap showing differential expression of Anoikis-related lncRNAs in tumor tissues and normal tissues. Red indicates high expression levels, while blue indicates low expression levels. **(E)** A general cross- validation curve of the paired likelihood deviance. **(F)** Elucidation for the LASSO coefficient profiles of the prognostic lncRNAs. **(G)** Forest plot highlighting the multiple Cox regression analysis results.

We conducted differential analysis to get 1,214 lncRNA-related differentially expressed genes between 88 OS tissues and 803 normal tissues with |log2FC| >1 and FDR <0.05. Then, we carried out Pearson’s correlation analysis to confirm 184 DEAKLncs with 27 anoikis-related genes from the GSEA database. Anokis-related genes and lncRNA were correlated and integrated into a network as shown in **Figures 1A, B**. First, 84 OS samples were randomly divided into two groups in a ratio of 1:1. Then, we constructed univariate Cox regression. The samples were divided into two groups based on the risk model, with more information about each group provided in **Table 1**. **Figure 1C** displays a heat map between OS tumor tissues and normal tissues and a nine-gene forest tree by univariate Cox regression (**Figure 1D**). The optimal value of ARLncs participating in the model construction by LASSO regression analysis was 6 (**Figures 1E, F** **)**. Through multi-Cox regression, the forest tree in **Figure 1G** was revealed. Lastly, four target genes (AC079612.1, MEF2C-AS1, SNHG6, and TBX2-AS1) were identified through uni-Cox regression, LASSO regression, and multi-Cox regression analyses to construct the prognostic model of both risk groups in OS patients, with three lncRNAs (MEF2C-AS1, SNHG6, and TBX2-AS1) considered as risk factors and one lncRNA (AC079612.1) as a protective factor for osteosarcoma patients. The coefficients for the four target genes can be found in **Table 2**. Through the formula for calculating the risk score, we can get the risk score for all samples by calculating them as follows: risk score = (-0.422 * AC079612.1exp) + (0.963 * MEF2C-AS1exp) + (0.619 * SNHG6exp) + (0.751 * TBX2-AS1exp).

**Table 1:** Clinical features of all osteosarcomas categorized into the training and validation cohorts.

Covariates	Type	Total	Train	Test	*P*-value
	Female	36 (42.86%)	20 (47.62%)	16 (38.09%)	0.5697
Gender	Male	47 (55.95%)	22 (52.38%)	25 (59.53%)	
	Unknown	1 (1.19%)		1 (2.38%)	
	≤14	44 (52.38%)	21 (50.00%)	23 (54.76%)	0.7365
Age	>14	39 (46.43%)	21 (50.00%)	18 (42.86%)	
	Unknown	1 (1.19%)		1 (2.38%)	
	Metastatic	21 (25.00%)	11 (26.19%)	10 (23.81%)	1
Metastasis	Non-metastatic	62 (73.81%)	31 (73.81%)	31 (73.81%)	
	Unknown	1 (1.19%)		1 (2.38%)	
	Arm/hand	6 (7.14%)	4 (9.53%)	2 (4.76%)	0.716
Tumor site	Leg/foot	75 (89.29%)	37 (88.09%)	38 (90.48%)	
	Pelvis	2 (2.38%)	1 (2.38%)	1 (2.38%)	
	Unknown	1 (1.19%)		1 (2.38%)	

**Table 2:** Optimal prognostic risk signature of four lncRNA by multivariate Cox regression analysis.

lncRNA	Coefficient	HR	HR.95L	HR.95H	P-value
AC079612.1	-0.422	0.656	0.439	0.980	0.040
MEF2C-AS1	0.963	2.620	1.364	5.035	0.004
SNHG6	0.619	1.856	1.108	3.114	0.019
TBX2-AS1	0.751	2.120	1.366	3.290	0.001

**Figure 2:**
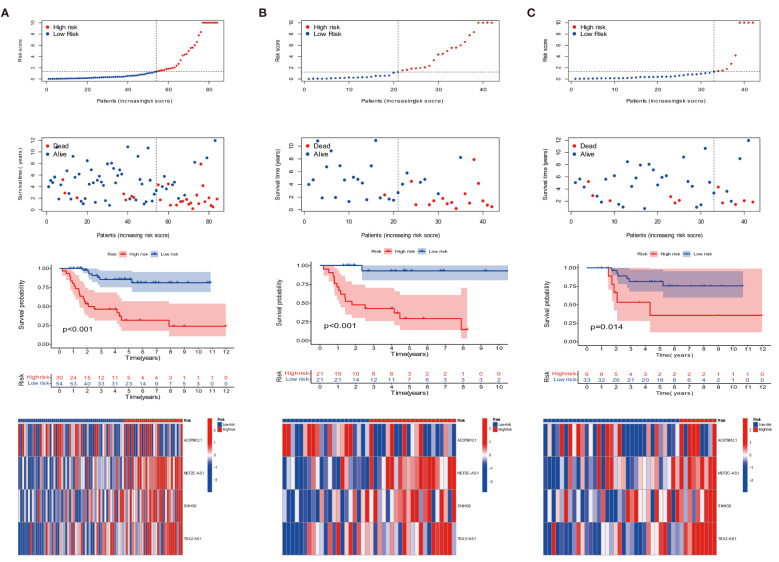
Prognosis of the risk model in the entire, training, and testing sets. **(A–C)** Survival time, survival status, Kaplan–Meier survival curves of patients with OS, and heat map in the expression of four lncRNAs in the entire, training, and testing sets, respectively.

**Figure 3:**
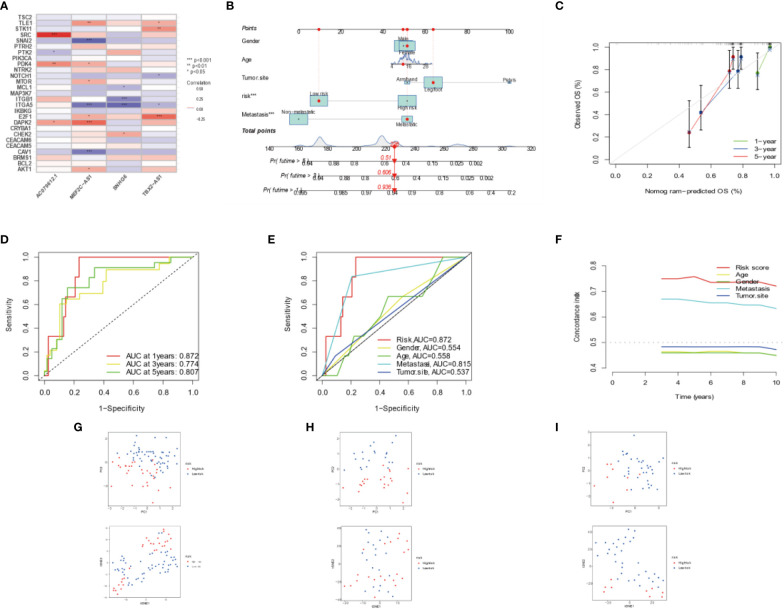
Combination of four DEARLncs signature and clinical features, analysis of PCA and t-SNE. **(A)** Relationship between four target genes and Anoikis-related lncRNAs in the entire cohort. **(B)** Nomogram based on age, gender, tumor site, metastasis, and risk in the TARGET database. **(C)** Calibration curves illustrated the consistency between predicted and observed 1-, 3-, and 5-year survival rates in OS patients depending on the prognostic nomogram. **(D, E)** The ROC curves for 1, 3, 5 years and clinical survival in the TARGET cohort. **(F)** The C-index was used to determining the prognostic accuracy of risk scores and addition clinical factors in entire cohort. **(G–I)** The PCA and t-SNE in entire, training, testing sets, respectively.

We compared the expression of four target anoikis-related lncRNAs in the low- and high-risk groups to evaluate the prognostic capability of the risk model, the distribution of risk score, and the survival status of the four ARLncs between risk subgroups in the whole group, training group, and test group (**Figures 2A–C**). In a Kaplan–Meier survival study of three groups, the prognosis of the low-risk group was statistically significantly better than that of the high-risk group. **Figure 3A** displays the relationship between the four strong prognostic lncRNAs and the 27 anoikis genes throughout the whole TARGET OS cohort.

The prognosis of OS patients was predicted using a nomogram that contained clinical–pathological factors and risk scores at the 1-, 3-, and 5-year intervals (**Figure 3B**). According to the calibration curves, the actual overall survival rates and the anticipated survival rates at 1, 3, and 5 years were in good agreement (**Figure 3C**). The AUCs of the 1-, 3-, and 5-year survival rates for the ARLncs signature were 0.872, 0.774, and 0.807, respectively (**Figure 3D**). When combined with clinical data, the AUCs of risk, gender, age, metastasis, tumor site, and the AUCs of risk were the best in predicting overall survival in OS patients; metastasis ranked second (**Figure 3E**). The C-index was used to determining the prognostic accuracy of risk scores and addition clinical factors in entire cohort (**Figure 3F**). The expression levels of the OS prognostic ARLncs were used in the risk model construction of the entire, training, and test cohorts, respectively, and were shown to significantly identify patients in the low- and high-risk groups, which can demonstrate the accuracy of the model. This was displayed by PCA and t-SNE (**Figures 3G–I** **)**. Above all, the prognostic model can be well predicted in osteosarcoma patients.

### Independent prognostic signature

**Figure 4:**
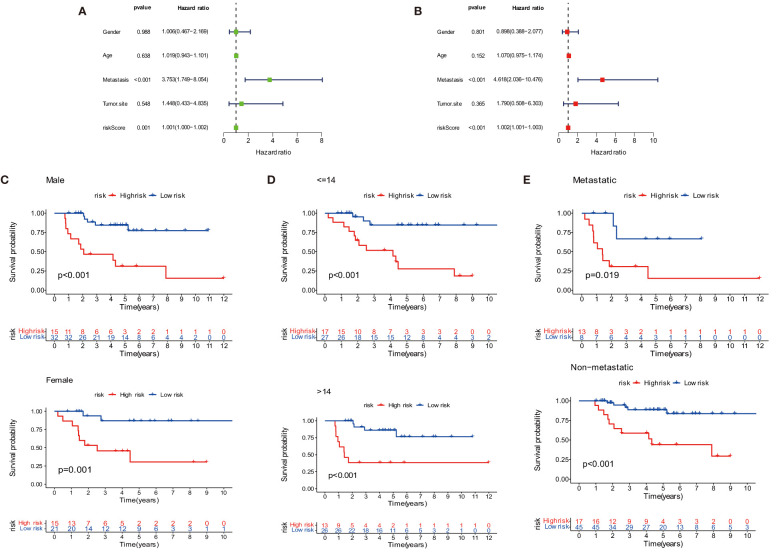
Prognosis of the anoikis-related lncRNA signature in osteosarcoma patients. **(A)** Univariate COX regression analysis. **(B)** Multivariate COX regression analysis. **(C)** Survival curve of gender. **(D)** Survival curve of age. **(E)** Survival curve of metastasis.

**Figure 5:**
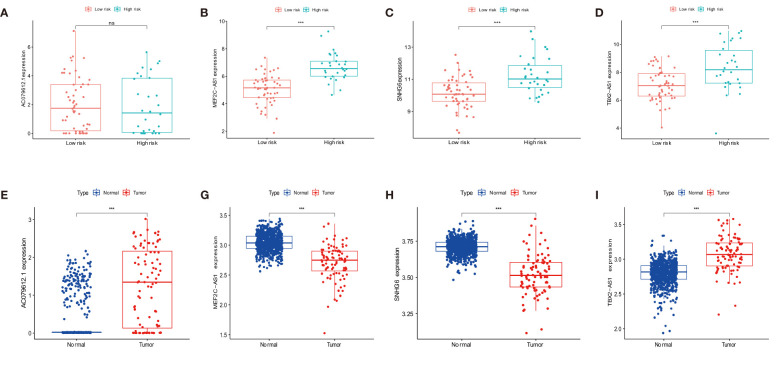
Differential expression of target genes in different osteosarcoma risk and tumor and normal tissues for the expression of AC079612.1, MEF2C-AS1, SNHG6, and TBX2-AS1 in various risk groups **(A–D)** and different tissue types **(E–I)**. "ns" P > 0.05, *** P < 0.001.

The overall survival-related variable between risk score and clinical factors was analyzed using the univariate Cox regression method (**Figure 4A**). The outcome demonstrated a statistically significant difference between metastatic status and risk score. The risk score and metastatic status were found to be independent risk factors affecting the prognosis of OS when we utilized multivariate Cox regression to analyze the risk score and clinical factors at the same time (**Figure 4B**). In order to compare the survival differences of the low- and high-risk groups in various clinical subgroups, we divided the OS patients into groups based on gender, age (≤14 and >14), and metastasis. The results showed that OS patients had a better clinical prognosis in the low-risk group than in the high-risk group among different genders, ages, and metastatic statuses (**Figures 4C–E**). Therefore, the risk model constructed by the four anoikis-related lncRNAs in the low-risk group would affect the survival prognosis of OS patients, which, combined with metastasis, would be a novel prognosis signature. The expression levels of anoikis-related lncRNAs (AC079612.1, MEF2C-AS1, SNHG6, and TBX2-AS1) showed differences in risk subgroups (**Figures 5A–D**) and different tissue types (**Figures 5E–I**). In the risk subgroups, the expression level of AC079612.1 was lower in the high-risk group than in the low-risk group, acting as a protective factor (**Figure 5A**), but other genes (MEF2C-AS1, SNHG6, and TBX2-AS1) were higher in the high-risk group (**Figures 5B–D**). In contrast, the higher differential expression level of AC079612.1 in tumor tissues was obvious compared with normal tissues (**Figure 5E**).

### Functional enrichment analysis

**Figure 6:**
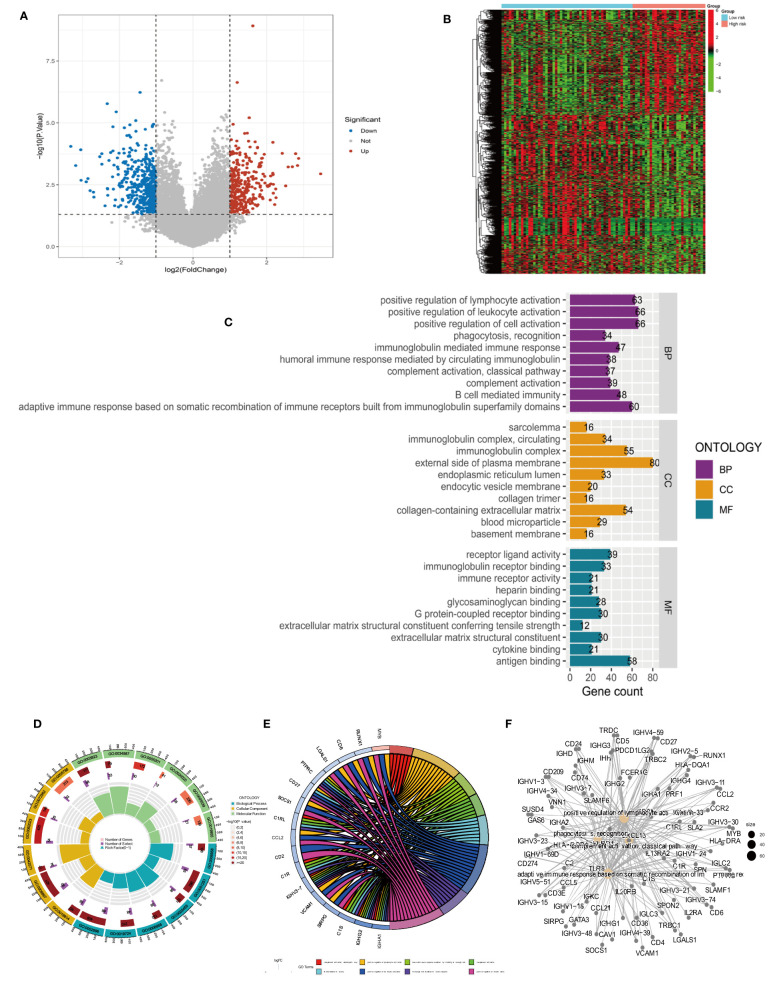
Differential analysis and GO functional enrichment analysis of the TARGET database in the high- and low-risk groups. **(A)** Volcano plot of differentially express(DEGs): red for high expression and blue for low expression. **(B)** Heat map of DEGs, with high expression in red and low expression in green. **(C)** Gene Ontology (GO) analysis for the DEGs between the high- and low-risk groups. **(D)** Circle plots for the analysis. **(E)** GO enrichment analysis of DEARLncs. **(F)** Network of biological processes in GO analysis.

**Figure 7:**
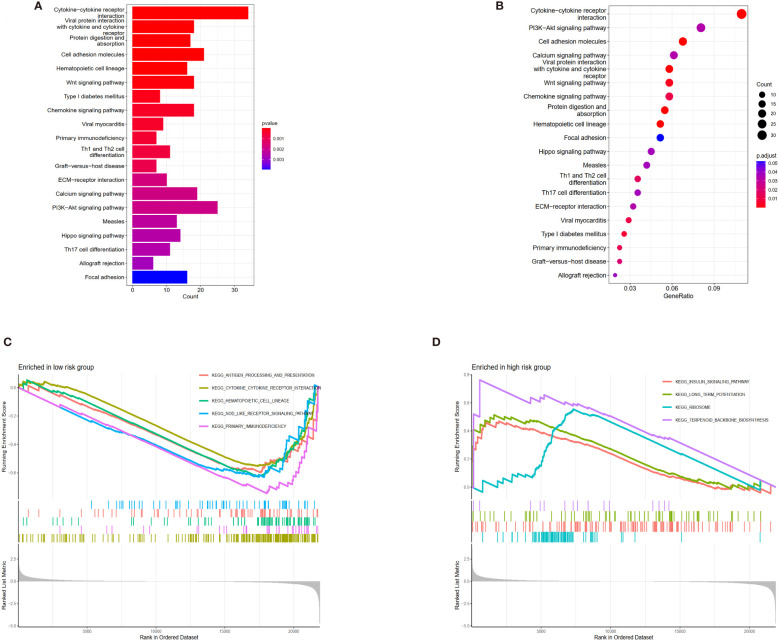
Kyoto Encyclopedia of Genes and Genomes (KEGG) and Gene Set Enrichment Analysis (GSEA) of different risk subgroups. **(A)** Bar plot of KEGG. **(B)** Bubble of KEGG. **(C)** Multiple pathways and functions were found to be enriched in low-risk anoikis-related lncRNA. **(D)** GSEA analysis in high-risk groups.

Differentially analyzing the genes in both risk groups helped identify the potential molecular mechanisms of the four-anoikis-related lncRNA signatures. A total of 833 DEGs were found with |logFC| >1 and a *P*-value <0.05. In total, 322 genes were upregulated, and 501 genes were downregulated among the DEGs. **Figures 6A, B**, respectively, display the volcano plots and heat map of anoikis-related lncRNAs with differential expression in the low- and high-risk groups. A GO analysis that we conducted revealed that the DEGs were primarily enriched in immune activation and immune response pathways. The GO bubble displayed a positive regulation of leukocyte activation in BP, exterior side of plasma membrane in CC, and antigen binding in MF (**Figure 6C**). **Figure 6D** shows the different function ratio of GO terms in GO circle. The GO chord showed that the positive regulation of lymphocyte activation and the positive regulation of cell activation were mainly enriched (**Figure 6E**). In the GO analysis, the network of biological processes is displayed in **Figure 6F**. According to the KEGG analysis, the top 22 KEGG terms in **Figures 7A, B** were primarily enriched in the cytokine–cytokine receptor interaction and PI3K-Akt signaling pathway. Overall, GO and KEGG analysis showed a strong link between anoikis and immunological status. To determine the potential process and pathway between the low- and high-risk groups, GSEA was carried out for the KEGG pathway enrichment analysis (**Figures 7C, D**). Most immune-related pathways, including antigen processing and presentation, cytokine–cytokine receptor interaction, hematopoietic cell lineage, Nod-like receptor signaling pathway, and primary immunodeficiency, were proven to exist in the low-risk group. On the other hand, several pathways were involved in OS development, including insulin signaling pathway, long-term potentiation, ribosome, and terpenoid backbone biosynthesis, and were mainly enriched in the high-risk group. Overall, the results showed that these tumor and immune-related pathways were regulated by the novel anoikis-related lncRNA signature. The microenvironment and immune infiltration of tumor in both risk groups

**Figure 8:**
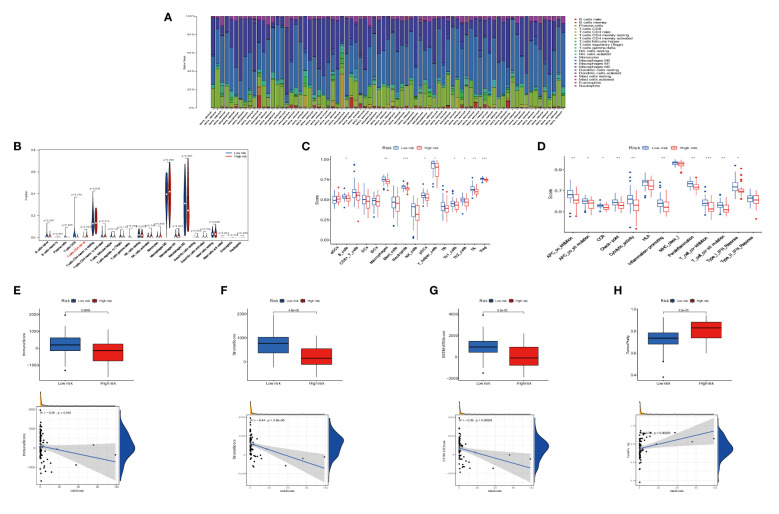
Immune signature in the low- and high-risk groups. **(A)** Proportion of 22 immune cell types in osteosarcoma risk groups. **(B)** Expression of immune cells between two groups. **(C)** The differential expression of cell infiltration between risk groups is based on the ssGSEA scores. **(D)** Immune functional difference between risk groups. **(E–H)** Differential expression of tumor microenvironment scores (immune scores, stromal scores, ESTIMATE scores, and tumor purity) between risk groups. * P < 0.05, ** P < 0.01, *** P < 0.001.

**Figure 9:**
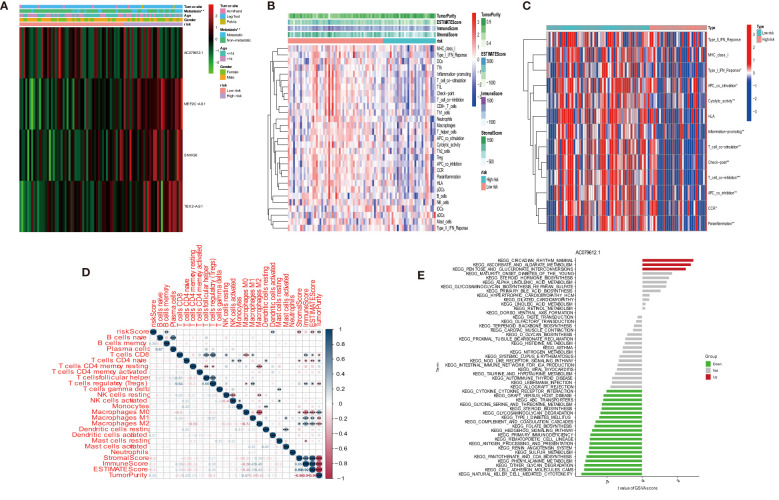
Distribution and visualization of immune status. **(A)** Heat map and the clinical characters of the two groups (***P < 0.001). **(B)** Heat map for immune status based on ESTIMATE and ssGSEA among two risk subgroups. **(C)** Heat map visualizing the infiltrated immune cells in the low- and high-risk groups. **(D)** Correlation between immune cells and riskScores, ImmuneScore, StromalScore, ESTIMATEScore, and TumorPurity. *P < 0.05, **P < 0.01, ***P < 0.001. **(E)** Count of pathway by AC079612.1 gene in GSVA.

We investigated the infiltration of 22 immune cell types in OS patients using the CIBERSORT algorithm. Most immune cell filtration were naive CD4 T cells, macrophages M0, M1, and M2, and activated dendritic cells (**Figure 8A**). According to risk groups, the expression of naïve CD4 T cells was higher in the high-risk group than in the low-risk group (*P* = 0.004); other immune cells did not have statistical significance in both risk groups (**Figure 8B**). The levels of immune cell infiltration were evaluated using the single sample gene set enrichment analysis (ssGSEA) based on the TARGET dataset (31). The box plot revealed that immune cell infiltration had lower scores in high-risk groups than in low-risk groups. However, the aDC infiltration had a higher score in the high-risk subgroup (**Figure 8C**). The most functional pathways including APC_co_inhibition, Check-point, Cytolytic_activity, T_Cell_co-inhibition, and T_Cell_co-stimulation were mainly enriched in the low-risk group (**Figure 8D**). We further analyzed the relationship between immune cell infiltration and risk score to investigate the function of anoikis-related lncRNAs in the OS immune microenvironment. We evaluated the ESTIMATEScore, ImmuneScore, and StromalScore, which were higher in the low-risk group than in the high-risk group. Meanwhile, the scores showed a negative correlation with riskScore (**Figures 8E–G**). In contrast to the high-risk group, TumorPurity was lower in the low-risk group. Similar to the box plot, the correlation was positive with riskScore (**Figure 8H**). When combined with clinical data, the heat map showed that the expression of three anoikis-related lncRNAs (MEF2C-AS1, SNHG6, and TBX2-AS1) was higher in the high-risk group compared with the low-risk group, while AC079612.1 was expressed at a lower level in the high-risk group. At the same time, statistical significance (*P* < 0.001) revealed that the high-risk group had a strong correlation with osteosarcoma metastasis (**Figure 9A**). The heat map shows the infiltration of immune cells in different risk groups combined with the immune microenvironment—for example, the expression of Type_II_IFN_Response was higher in the high-risk group, and there was a higher score in Stromalscore, ImmuneScore, ESTIMATEScore, and TumorPurity (**Figure 9B**). We know that the immune-related functions were significantly downregulated in the high-risk group (**Figure 9C**). Regarding the correlation between tumor microenvironment infiltration and riskScores, ImmuneScore, Stromalscore, ESTIMATEscore, and TumorPurity in the risk groups, we could see that riskScore was related with B cell memory, activated dendritic cells, Stromalscore, ESTIMATEscore, and TumorPurity (**Figure 9D**). The pathways in GSVA were regulated by the AC079612.1 gene, such as the pathways including kegg_circadian_rhythm_mammal and kegg_ascorebate_and_aldarate_metabolism, which were enriched in the Up group (**Figure 9E**).

### Correlation between immune cells and target genes, drug sensitivity, and immune checkpoint molecule expression analysis

**Figure 10:**
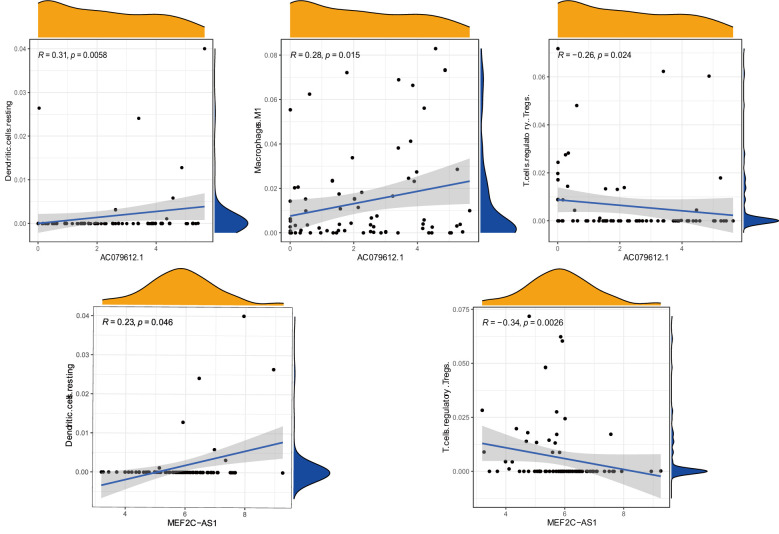
Correlation between immune cells (Dendritic.cells, T.cells.regulatory, Macrophages.M1, T.cells.regulatory, and Dendritic.cells) and target genes (AC079612.1 and MEF2C-AS1).

**Figure 11:**
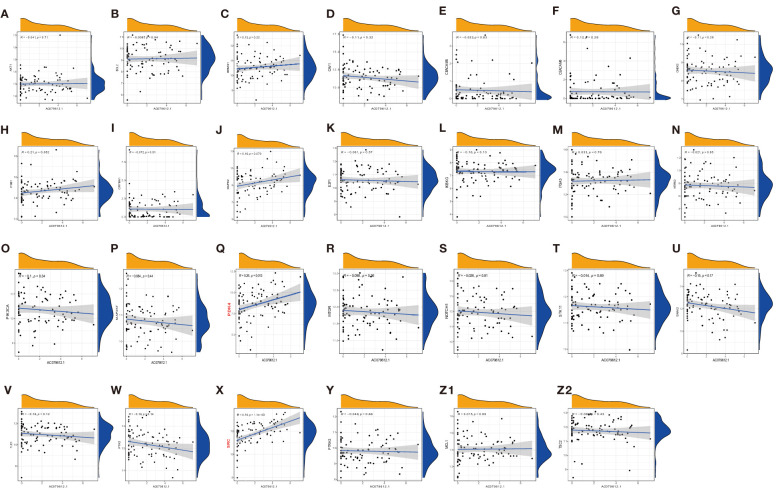
Pearson correlation analysis of AC079612.1 with anoikis-related genes (**A**–**Z2**: CRYBA1, ITGB1, MAP3K7, NOTCH1, CEACAM6, IKBKG, CAV1, MCL1, BRMS1, SNAI2, ITGA5, MTOR, DAPK2, PIK3CA, PTRH2, PDK4, TLE1, TSC2, E2F1, CHEK2, CEACAM5, PTK2, AKT1, NTRK2, STK11, SRC, and BCL2).

**Figure 12:**
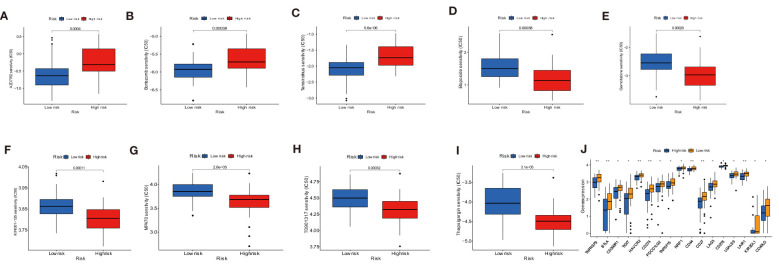
Drug sensitivity analysis and immune checkpoint molecules expression analysis. **(A–I)** Sensitivity performance of 9 drugs in the high-risk and low-risk groups. **(J)** Differential expression of immune checkpoint genes between low- and high-risk groups. * P < 0.05, ** P < 0.01.

Under a statistical significance, Dendritic cells resting and Macrophages M1 had a positive correlation with AC079612.1, and T cells regulatory (Tregs) was shown to be negatively correlated with AC079612.1. Resting dendritic cells were positively correlated with MEF2C−AS1 but had a negative correlation with Tregs (**Figure 10**). Based on the above-mentioned data, AC079612.1 gene might be a good biomarker to predict the therapy and diagnose osteosarcoma. Therefore, we explored the correlation between AC079612.1 and 27 anoikis-related genes by conducting Pearson’s correlation analysis. The results showed that AC079612.1 expression was positively correlated with the expression of PDK4 and SRC genes (|*R*| > 0.4, *P* < 0.05) (**Figures 11A–Z2**). The drug sensitivity analysis revealed that AZD7762, bortezomib, etoposide, gemcitabine, KIN001-135, MP470, T0901317, temsirolimus, and thapsigargin (*P* < 0.001) all showed statistically significant sensitivity in both risk groups. AZD7762, bortezomib, and etoposide had more drug sensitivity in both risk groups, while patients in the high-risk group were more sensitive to gemcitabine, KIN001-135, MP470, T0901317, temsirolimus, and thapsigargin than those in the low-risk group (**Figures 12A–I**). The different drugs were sensitive in the treatment of OS patients by drug sensitivity analysis. Given the importance of checkpoint-based immunotherapy, we noted that the expression of TNFRSF9, BTLA, CD200R1, TIGIT, HAVCR2, CD274, PDCD1LG2, TNFSF15, NRP1, CD44, CD27, LAG3, CD276, LGALS9, LAIR1, KIR3DL1, and CD40LG was higher in the low-risk group than in the high-risk group (**Figure 12J**), showing that OS patients could better respond to immunotherapy.

## Discussion

The majority of occurrences of osteosarcoma, the most common malignant bone tumor, are seen in children and young people between the ages of 10 and 30 (32). Despite the fact that standard treatments, such as a series of comprehensive treatments including surgery and chemotherapy, increased the survival rates, certain patients’ odds of surviving with OS were not good (33). Drug resistance during the therapy process and OS metastases caused the treatment effects to be unsatisfactory (34). Therefore, it is imperative to find reliable prognostic indicators for treating OS patients.

Anoikis, which prevents shed cells from sticking to a new substrate in the wrong location and restricting the organism’s growth, is an essential defense mechanism (35). According to certain studies, the intrinsic and extrinsic mechanisms must both be present for anoikis apoptosis to take place (36). While mitochondria are important to regulate apoptosis, a number of intracellular signals, such as DNA damage, can also cause anoikis (37). This disorder in anoikis execution, which promotes tumor cell invasion and migration, the organ’s growth through metastasis, and the establishment of drug resistance, may be present in cancer cells (38–40). There were increasing studies that showed anoikis resistance and pulmonary metastasis in the OS microenvironment by altering related signal pathways (41).

Additionally, a growing amount of data suggests that lncRNA plays critical roles in controlling the development of various cancers (42). The prognosis of tumor patients was predicted using a variety of novel lncRNA models based on anoikis (19, 43). Currently, there is no research to report on the study of both anoikis-related lncRNA and the clinical characteristics of OS patients. Hence, finding the anoikis-related lncRNAs as the prognosis markers of osteosarcoma is essential. In this study, we conducted a comprehensive analysis of the target lncRNAs and the related immune microenvironment to explore the potential molecular mechanisms of OS progression.

In this study, the expression levels of the ARGs in OS and normal tissues were investigated to assess the prognostic efficacy of anoikis-related lncRNAs in OS patients. Then, the uni-Cox regression analysis was used to identify nine prognostic ARLncs. Four important ARLncs, including AC079612.1, MEF2C-AS1, SNHG6, and TBX2-AS1, were screened using the LASSO Cox and Multiple Cox regression analysis for building the optical model, and a four-ARLncs signature for osteosarcoma was effectively constructed. Of the four anoikis-related lncRNAs, we discovered that AC079612.1 was a protective factor with a lower expression in the high-risk subgroup of osteosarcoma. Patients at high risk have a significantly lower rate of survival than patients at low risk. The validation cohort’s prognosis also demonstrated the model was well. Additionally, a great nomogram for predicting survival rates was developed using the risk scores and additional clinical factors (such as gender, age, metastasis, and tumor site). In conclusion, these results demonstrated the four-ARLncs signature’s applicability to other cohorts and supported its strong predictive significance in our study’s osteosarcoma patients. The tumor microenvironment, which played a significant role in the treatment of OS, was identified as one of the most critical factors affecting immunotherapy (44, 45). Currently, immune-related therapy has become an increasingly important strategy for OS patients (46, 47). We explored functional enrichment analysis using GO, KEGG, and GSEA to investigate the anoikis-related lncRNAs in OS patients. The pathways of antigen processing and presentation, cytokine–cytokine receptor interaction, hematopoietic cell lineage, and Nod-like receptor signaling pathway were found in related immune-mechanisms of OS. Next, the relationship between the anoikis-related lncRNAs signature and the immune status of osteosarcoma was then analyzed. The tumor microenvironment in OS was explored using the ESTIMATE, ssGSEA, and CIBERSORT algorithms. Finally, we identified the correlation between risk scores and immune cells and the association of target ARLncs with anoikis-related genes. Of course, drug sensitivity and immune checkpoint studies were conducted to explore the effectiveness of drug therapy and immunotherapy in both risk groups.

Anoikis-related lncRNAs are closely related to the mechanism of tumorigenesis, but the relationship between osteosarcoma and ARLncs is not known. Therefore, it is of great potential value to study the mechanism and treatment of osteosarcoma from the point of view of ARLncs. Through comprehensive analysis, four ARLncs genes and related immune pathways were identified, which is of great help in understanding the mechanism of osteosarcoma at the genetic level. The drug sensitivity analysis provides a new theoretical support for clinicians treating osteosarcoma with drugs.

However, there are some limitations in this study. First, due to the gene expression derived from the public TARGET database, the samples of osteosarcoma are small. Future work should focus on developing larger samples to improve the accuracy of the results. Second, there were only 84 clinical data added to the database; if possible, the clinical data can be collected in hospitals to increase the abundance of clinical data, and the data on tumor stage in OS was lacking. Third, the other testing dataset is needed to assess the value of the risk score model. Fourth, there is absence of a corresponding experiment with target genes to verify the expression and effectiveness of genes *in vivo* in clinical trials. Finally, the underlying mechanism of four ARLncs (AC079612.1, MEF2C-AS1, SNHG6, and TBX2-AS1) regulating the prognosis of OS patients should be explored further. These drawbacks will be included in our follow-up plans and addressed. For the small sample size of osteosarcoma, we will collect clinical samples for further research in the future. At the same time, if conditions permit, we will also collect osteosarcoma samples for experimental verification, thereby increasing the reliability of the conclusion.

## Conclusion

The risk model would be constructed using four ARLncs (AC079612.1, MEF2C-AS1, SNHG6, and TBX2-AS1). We found that the four ARLncs can influence OS’s immunologic microenvironment. Nine drugs and immunotherapy can be identified as potential therapeutical schemes for OS. These results of the study are helpful to increase the therapeutic effectiveness and improve the general survival rate of individuals with osteosarcoma.

## Data availability statement

The datasets presented in this study can be found in online repositories. The names of the repository/repositories and accession number(s) can be found below: TCGA, TARGET-OS, phs000218.

## Author contributions

J-SZ constructed the scheme and collected the data of the study. J-SZ drafted the manuscript, and R-SP revised the manuscript. Funding acquisition was from X-BT. All authors contributed to the article and approved the submitted version.
